# Enriched Environment Shortens the Duration of Action Potentials in Cerebellar Granule Cells

**DOI:** 10.3389/fncel.2019.00289

**Published:** 2019-07-16

**Authors:** Abdelmoneim Eshra, Petra Hirrlinger, Stefan Hallermann

**Affiliations:** ^1^Medical Faculty, Carl-Ludwig-Institute for Physiology, Leipzig University, Leipzig, Germany; ^2^Medical Faculty, Medizinisch-Experimentelles Zentrum, Leipzig University, Leipzig, Germany

**Keywords:** enriched environment, action potential, granule cell, cerebellum, electrophysiology

## Abstract

Environmental enrichment for rodents is known to enhance motor performance. Structural and molecular changes have been reported to be coupled with an enriched environment, but functional alterations of single neurons remain elusive. Here, we compared mice raised under control conditions and an enriched environment. We tested the motor performance on a rotarod and subsequently performed whole-cell patch-clamp recordings in cerebellar slices focusing on granule cells of lobule IX, which is known to receive vestibular input. Mice raised in an enriched environment were able to remain on an accelerating rotarod for a longer period of time. Electrophysiological analyses revealed normal passive properties of granule cells and a functional adaptation to the enriched environment, manifested in faster action potentials (APs) with a higher depolarized voltage threshold and larger AP overshoot. Furthermore, the maximal firing frequency of APs was higher in mice raised in an enriched environment. These data show that enriched environment causes specific alterations in the biophysical properties of neurons. Furthermore, we speculate that the ability of cerebellar granule cells to generate higher firing frequencies improves motor performance.

## Introduction

Environmental enrichment (EE) refers to refined conditions for housing animals, which result in enhanced motor, social, sensory and cognitive performances (Nithianantharajah and Hannan, 2006). In the 1940s, Donald Hebb used EE and showed that rats, which were raised in his home, had superior problem solving abilities compared to laboratory-raised rats (Hebb, 1947, 1949). In addition, EE has been reported to improve motor performance when checked with assays such as rotarod, eyeblink conditioning, grid walking, rope suspension, footfault, and walk initiation tests (Madroñal et al., 2010; Horvath et al., 2013; Lee et al., 2013).

On the anatomical level, EE leads to thicker regions in the cerebellar cortex (Diamond et al., 1966) and altered dendritic and spine morphology (Volkmar and Greenough, 1972; Restivo et al., 2005). EE robustly induces neurogenesis in the hippocampus (Kempermann et al., 1997) and cell proliferation in the amygdala (Okuda et al., 2009) as well as gliogenesis, manifested in an increase in the number of new astrocytes in the visual cortex (Sirevaag and Greenough, 1987), the motor cortex (Ehninger and Kempermann, 2003) and the hippocampus (Kronenberg et al., 2007). Furthermore, EE increases the number of myelin-forming oligodendrocytes (Szeligo and Leblond, 1977; Sirevaag and Greenough, 1987) and the number of myelinated fibers in the cerebral white matter (Yang et al., 2013).On the molecular level, EE has been extensively studied. A change in the expression level of many genes involved in neuronal structure, synaptic plasticity, and neurotransmission have been reported (Rampon et al., 2000; Barak et al., 2013). Moreover, the expression levels of brain-derived neurotrophic factor (BDNF) and nerve growth factor (NGF) were found to be increased in association with EE (Torasdotter et al., 1998; Rossi et al., 2006).

Granule cells are the most abundant neurons in the brain (Williams and Herrup, 1988), representing the input layer, which translates mossy fiber signals into parallel fiber signals that project to Purkinje cells (Eccles et al., 1967). Granule cells seem to have various functional roles for sensory processing (Chadderton et al., 2004), locomotion (Powell et al., 2015), and reward predictions (Wagner et al., 2017). To gain a better understanding of the improvement of motor performance induced by EE on a cellular level, we here focused on the biophysical properties of granule cells in a specific region of the cerebellum, lobule IX, which receives vestibular sensory signals (Barmack, 2003) and is involved in motor tasks such as the rotarod test (Ruediger et al., 2011). We found that EE changed fundamental biophysical parameters of granule cells, such as the duration of action potentials.

## Materials and Methods

### Animals and Housing

**FIGURE 1:**
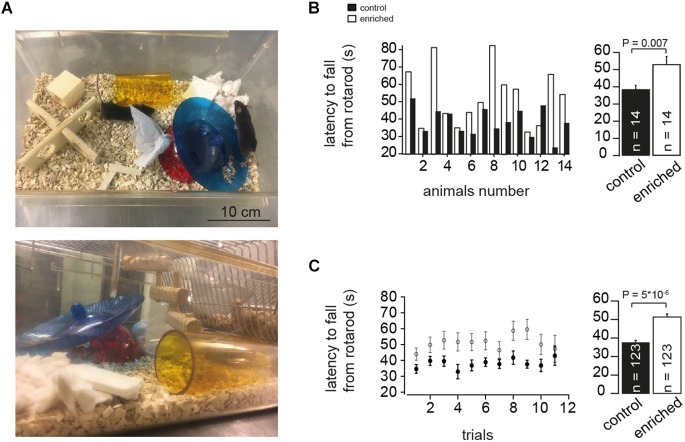
Enriched environment improves motor performance. **(A)** Example photographs of EE cage showing different items placed during the housing of the mice. **(B)** Left: Bar graphs showing comparison of the absolute latency on the rotarod per animal (animal-to-animal basis). Right: Bar graph of the average latency on the rotarod assessment test for EE and control mice, *n* = 14 and 14 mice, respectively; Student’s *t*-test was used to measure statistical significance, *P_t_*_-test_ = 0.007. **(C)** Left: Bar graphs showing comparison of the absolute latency on the rotarod per trial number (trial-to-trial basis). Right: Bar graph of the grand average of the latency on the rotarod, *n* = 123 and 123 trials, for control and EE mice, respectively; *P_t_*_-test_ = 5 × 10^-6^.

Mice were bred and treated in accordance with the German Protection of Animals Act and with the guidelines for the welfare of experimental animals issued by the European Communities Council Directive. Mice were housed in either EE or control conditions from birth (pre-weaning) until the age of P70-80 before testing. The EE cages were designed to be bigger (height: 140 and 150 mm, for control and EE, respectively; bottom: 252 × 167 and 427 × 267 mm, for control and EE, respectively) and contained climbing ladders, plastic tubes, tunnels and small boxes as well as a variety of other toys like igloos and saucer wheels (Figure 1A). Groups of mice designated to the EE cohort were housed in larger groups (∼5 and ∼9, for control and EE, respectively) to allow more social interactions. The configuration of the EE was changed approximately twice per week by rearranging the position of the toys and by adding and removing toys to provide novelty, complexity, and different opportunities for learning. Both mice groups, EE and controls, were housed under an appropriate temperature (21–23°C) and humidity (40–60%) in a controlled atmosphere with a 12 h/12 h light/dark cycle, and had free access to both water and food.

### Preparation and Electrophysiology

Acute cerebellar slices were prepared from mature (P70–80) C57BL/6 mice of either sex. Mice were lightly anaesthetized with isoflurane and killed rapidly by decapitation. The cerebellar vermis was removed quickly and then placed in a chamber filled with ice-cold extracellular solution. Parasagittal slices of 300 μm were cut using a Leica VT1200 microtome (Leica Microsystems, Wetzlar, Germany), transferred to an incubation chamber at ∼35°C for 30 min, and subsequently stored at room temperature. The extracellular solution for slice preparation, storage, and electrophysiological recordings contained (in mM) the following: NaCl 125, NaHCO_3_ 25, glucose 20, KCl 2.5, CaCl_2_ 2, NaH_2_PO_4_ 1.25 and MgCl_2_ 1 [310 mOsm, pH 7.3 when bubbled with Carbogen (5% (vol/vol) O_2_ and 95% (vol/vol) CO_2_)].

Cerebellar granule cells were visualized after mounting a slice into a recording chamber placed on the stage of a Nikon upright microscope equipped with infrared differential interference contrast. Slices were continuously superfused with extracellular solution and the temperature in the center of the recording chamber was set to 36°C using a TC-324B perfusion heat controller (Warner Instruments, Hamden, CT, United States). Patch pipettes were pulled from borosilicate glass (Science Products, Hofheim, Germany) using a DMZ Puller (Zeitz-Instruments, Munich, Germany). Patch pipettes had open-tip resistances of 6–8 MΩ (when filled with intracellular solution). The intracellular solution contained the following (in mM): K-gluconate 150, NaCl 10, K-HEPES 10, MgATP 3 and Na-GTP 0.3 (300–305 mOsm, pH adjusted to 7.3 with KOH). Whole-cell patch-clamp recordings from granule cells were made using a HEKA EPC10/2 amplifier (HEKA Elektronik, Lambrecht, Germany) operated by the corresponding software PatchMaster (HEKA Elektronik), running on a personal computer. Recordings were performed in the middle region of the granule cell layer of lobule IX of the cerebellar vermis. Measurements were corrected for a liquid junction potential of +13 mV. Series resistance was typically <40 MΩ. Experiments with series resistance >40 MΩ were excluded. Action potentials were evoked in current-clamp mode by injecting brief current pulses (amplitude 10–500 pA; duration 300 ms). Recordings from neurons of mice raised in an enriched environment and the corresponding control, of the same gender, were done in the same day in an interleaved manner.

### Rotarod Test

Before testing began, mice were given a trial in order to familiarize them with the procedure. Mice were placed on the rotarod (Panlab, Harvard apparatus) at a constant acceleration from 4 to 40 rounds per minute for a total of 120 s so that the longer the mouse remained on the rod, the faster it had to move to maintain balance. Each time the mouse fell, it was immediately returned to the rod and the process was restarted for ∼10 trials per mouse. EE and control mice were compared on an animal-to-animal basis by averaging the latencies per mouse (Figure 1B) and on a trial-to-trial basis by averaging the latencies per trial number (Figure 1C). Motor assessment of each EE mouse and the corresponding control, having the same gender, was done on the same day, and both were later sacrificed to be used for electrophysiological recordings.

### Analysis of Action Potential Parameters

Data were analyzed using custom-made procedures in Igor Pro software (WaveMetrics, Tigard, OR, United States). The parameters of the action potentials (APs) were determined in the trace at current threshold, the trace with 60-pA-current injection, and the trace with most APs elicited. In each of these three traces the analysis was restricted to the first AP, average of the first five APs at the beginning of the current injection, and average of all APs. In traces with less than five APs, the value for the first five APs was the average of those up to four APs. In four and one out of 299 cells in control and EE mice, respectively, the current threshold was above 60 pA, and accordingly the numbers of cells for the 60-pA-current trace were 295 and 298 for control and EE mice, respectively. The half duration of AP was measured at half maximum amplitude. The amplitude was measured from threshold to peak. The threshold was defined as the membrane voltage at which the first derivative exceeded 100 V/s. Action potentials with a peak smaller than -20 mV, an amplitude smaller than 20 mV, and a half duration smaller than 50 μs or larger than 500 μs were excluded. These exclusion criteria were chosen to ensure that only proper APs are analyzed. For example, the distribution of AP duration is well within the 50 and 500 μs borders (Supplementary Figure S2B).

### Statistics

Data are presented as mean ± SEM. To provide a simple measure of the statistical difference of the shown comparisons, the *P*-value of the Student’s *t*-test is provided for each bar graph (using Microsoft Excel or Igor Pro software). In addition, a four-way ANOVA test with the following four factors was used: (1) AP parameters (half-duration, amplitude, overshoot and threshold), (2) trace per cell (trace at current threshold, trace with 60 pA, and trace with maximal number of APs), (3) APs per trace (first AP, first five APs, and average of all APs), and (4) animal group (control and EE). For the comparison of control vs. EE mice (4th factor), the *P*-value was 0.0002. However, the significance level of the four-way ANOVA is most likely an overestimation, because the investigated parameters are not completely independent. We therefore focused on the *P*-values of the *t*-test (*P_t_*_-test_). For each of the four AP parameters, there was the same trend between control and enriched conditions in the various analyzed APs. In addition, we addressed the statistical significance of the difference of the AP half duration not only on a neuron-to-neuron but also on a mouse-to-mouse basis (i.e., the average value for each mouse). To address the significance of the correlations (Figure 4D and Supplementary Figures S3A,B), we provided the Pearson correlation coefficient (*R*_Pearson_) and the corresponding *P*-value (*P*_Pearson_) as well as the *P*-value of the Spearman rank correlation coefficient (*P*_SpearmanRank_). *T*-tests were calculated with Microsoft Excel and the other statistical tests with built-in functions of Mathematica 10 (Wolfram Research, Champaign, IL, United States).

## Results

### Enriched Environment Improves Motor Performance

To investigate the effect of continuous long-term EE (Figure 1A) on the motor performance of mice, we used the rotarod test. The performance of mice raised in an EE (referred to as EE mice in the following) was significantly better on the rotating rod than the corresponding control mice. This was manifested in a significantly longer latency to fall from the rotarod in the case of EE mice than for the corresponding controls, both for animal-to-animal overall performance (38.7 ± 2.1 and 53.2 ± 4.5 s for control and EE mice, *n* = 14 and 14 mice, respectively; *P_t_*_-test_ = 0.007; Figure 1B) and for trial-to-trial basis (38.1 ± 0.9 and 51.3 ± 1.4 s for control and enriched animals, *n* = 123 and 123 trials, respectively; *P_t_*_-test_ = 5 × 10^-6^; Figure 1C). This is consistent with previous findings where EE was reported to have a direct effect on motor functions when assessed with different motor coordination assays (Madroñal et al., 2010; Horvath et al., 2013; Lee et al., 2013). These data indicate that EE mice have improved motor capabilities as evaluated by the rotarod test.

### Action Potentials of EE Mice Have Shorter Half Duration

**FIGURE 2:**
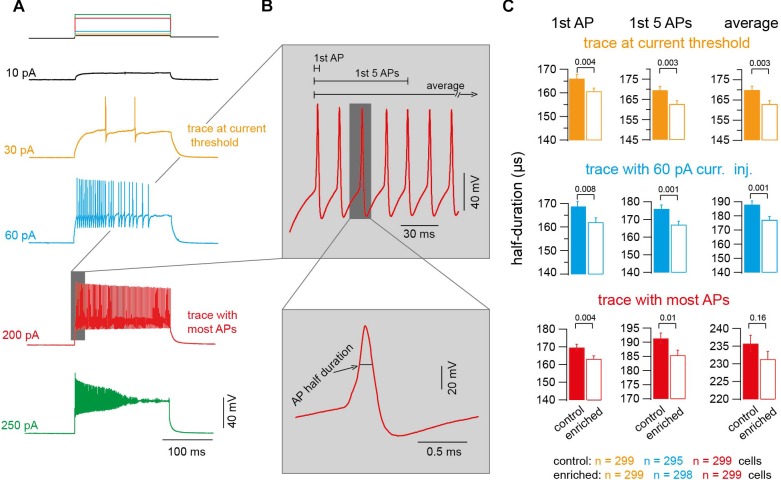
Action potentials of EE mice have shorter half duration. **(A)** Example voltage traces upon injecting current of increasing amplitudes (orange: trace at current threshold; cyan: trace with 60-pA-current injection; red: trace with maximum APs fired; green: trace with highest current injection and decrease in APs number). **(B)** Top: Magnification of the shaded part in panel **(A)** showing an example of APs. Bottom: Corresponding magnification showing a single AP. **(C)** Average AP half duration for control and EE mice (orange: trace at current threshold, *n* = 299 and 299 cells; cyan: trace with 60-pA-current injection, *n* = 295 and 298 cells; red: trace with maximum APs fired, *n* = 299 and 299 cells). From left to right, analysis of first AP, first five APs, and average of all APs. All the *P*-values shown are from Student’s *t*-test.

To test if and how these improvements in motor skills go along with alterations of the biophysical parameters of single neurons, we performed whole-cell current-clamp recordings from granule cells and measured the excitability of the neurons and the properties of the APs. We analyzed 90,750 APs, in 600 neurons in 30 mice, elicited by injection of depolarizing current of different amplitudes (10–500 pA; Figure 2A). Due to several reasons (like AP broadening and amplitude reduction), which can change the AP shape over the duration of injected current, and in order to properly compare the APs in both conditions, we focused on three traces to be representative for all the APs fired per neuron: (1) the trace with lowest current injection and at least one AP (current threshold), (2) the trace where APs were elicited upon 60 pA current injection, and (3) the trace where the highest number of APs appeared. Within each of these three traces, we analyzed the first AP, the average of the first five APs, and the average of all APs (Figure 2B). The half duration of the AP of neurons of EE mice was found to be significantly shorter than the half duration of APs of neurons of control mice (e.g., first AP in the traces at a current threshold: 166.1 ± 1.6 and 159.7 ± 1.5 μs for control and EE mice, *n* = 299 and 299, respectively; *P_t_*_-test_ = 0.002; Figure 2C). The analysis of other APs in other traces revealed similar results (Figure 2C). To further test the observed effect of EE on AP half duration, we compared the AP half duration on a mouse-to-mouse basis (i.e., the average value for each mouse). We found a tendency toward faster APs in EE compared to control mice (*P_t_*_-test_ ≈ 0.1; Supplementary Figure S1), indicating that a trend with a significance level of 10% exists even on a mouse-to-mouse basis. In order to test the robustness of our measurements, we compared the AP half duration of control mice of different ages from P20 to 1 year, and found that the AP half duration did not change significantly between different age groups (Supplementary Figure S2A), indicating that our technique allows precise and reliable determination of AP parameters. The AP seems to be a constant parameter throughout development between the age of P20 and 1 year. Despite this developmental constancy, our data demonstrate that the AP half duration of cerebellar granule cells of mice raised in an EE environment is shorter compared to the corresponding control mice.

### Alteration in Threshold, Overshoot, and Amplitude of Action Potentials Upon Enrichment

**FIGURE 3:**
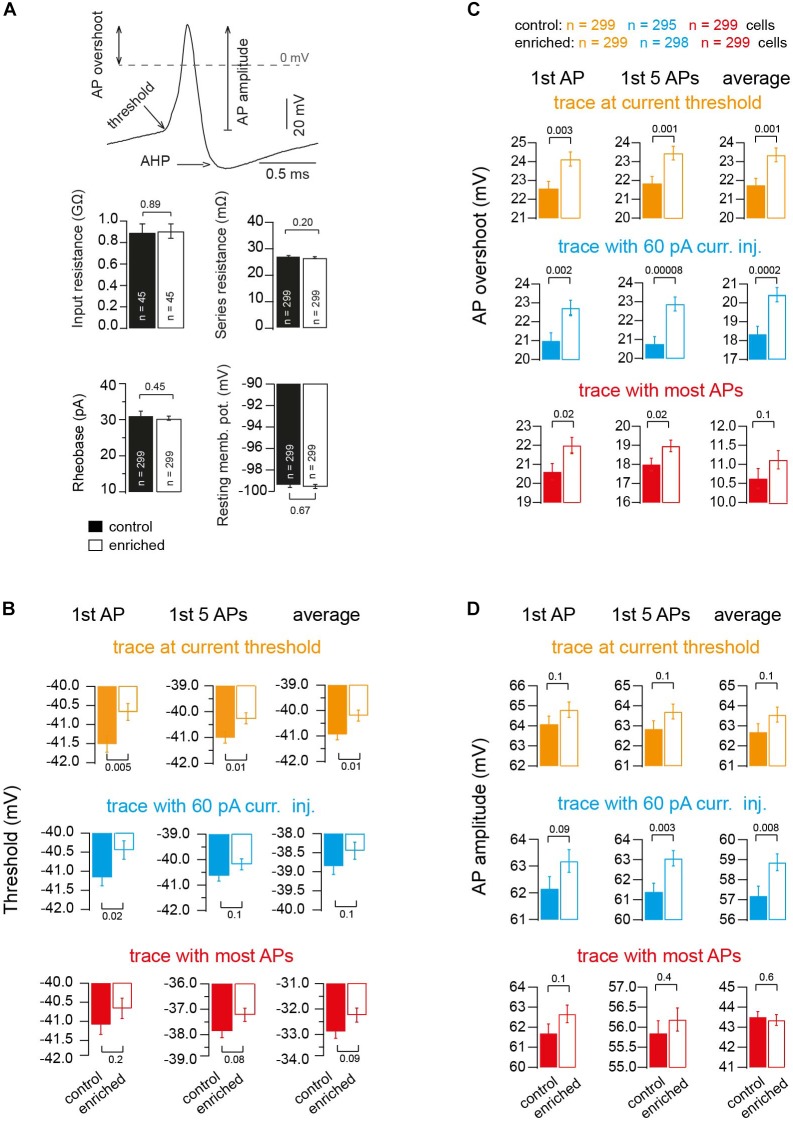
Alteration in threshold, overshoot and amplitude of action potentials upon enrichment. **(A)** Example of an AP illustrating the criteria of measuring AP threshold, AP overshoot and AP amplitude. Bar graphs showing the average of resting membrane potential, *n* = 299 and 299 cells; input resistance, *n* = 45 and 45 cells; rheobase, *n* = 299 and 299 cells; and series resistance, *n* = 299 and 299 cells, of neurons of control and EE mice, respectively. **(B)** Average voltage threshold of APs of neurons of control and EE mice (orange: trace at current threshold, *n* = 299 and 299 cells; cyan: trace with 60-pA-current injection, *n* = 295 and 298 cells; red: trace with maximum APs fired, *n* = 299 and 299 cells). From left to right, analysis of first AP, first five APs and average of all APs, respectively. **(C)** Corresponding average AP overshoot. **(D)** Corresponding average AP amplitude. All the *P*-values shown are from Student’s *t*-test.

To gain insight into the different biophysical properties of APs of EE mice, we first compared the input resistance by injecting current of low amplitude into a subset of granule cells (∼3) from each animal of either the EE or control groups, and observed no significant change (0.88 ± 0.08 and 0.90 ± 0.07 GΩ, for control and EE mice, *n* = 45 and 45, respectively; *P_t_*_-test_ = 0.89; Figure 3A). Other passive parameters of neurons were similar, too (series resistance: 27.4 ± 0.4 and 26.7 ± 0.4 MΩ, for control and EE mice, *n* = 299 and 299, respectively; *P_t_*_-test_ = 0.2; rheobase: 31.3 ± 1.1 and 30.4 ± 0.5 pA; *P_t_*_-test_ = 0.45; and resting membrane potential: -99.4 ± 0.3 and -99.5 ± 0.2 mV; *P_t_*_-test_ = 0.67; Figure 3A).

In order to gain more mechanistic insights, we focused on additional parameters of the APs and found that the threshold potential of the APs of EE mice had a more depolarized voltage than the APs of control mice (e.g., first AP in the traces at current threshold: -41.5 ± 0.21 and -40.67 ± 0.22 mV for control and EE mice, *n* = 299 and 299, respectively; *P_t_*_-test_ = 0.005). The analysis of other APs in other traces revealed similar results (Figure 3B). The maximum peak of the voltage overshoot reached during the AP was higher in EE compared to control mice (e.g., first AP in the traces at current threshold 22.58 ± 0.36 and 24.13 ± 0.37 mV, for control and EE mice, *n* = 299 and 299, respectively, *P_t_*_-test_ = 0.003). The analysis of other APs in other traces revealed similar results (Figure 3C). Furthermore, there was also a tendency for the absolute amplitude of APs to be higher in the case of EE mice compared to control mice (e.g., first AP in the traces at current threshold 64.09 ± 0.38 and 64.8 ± 0.38 mV, for control and EE mice, *n* = 299 and 299, respectively; *P_t_*_-test_ = 0.1). The analysis of other APs in other traces revealed similar results (Figure 3D).

Interestingly, when analyzing the after-hyperpolarizing (AHP) component of the APs, there was no significant difference between EE and control mice. For the trace at current threshold, the delay between the peak (maximum) of the AP and the peak (minimum) of the AHP was not different between EE and control mice groups (402.6 ± 3.3 and 401.9 ± 3.4 μs for control and EE mice, *n* = 299 and 299, respectively; *P_t_*_-test_ = 0.89), and the AHP voltage did not change either (e.g., first AP in the traces at current threshold: –64.9 ± 0.2 and –64.8 ± 0.2 mV for control and EE mice, *n* = 299 and 299, respectively; *P_t_*_-test_ = 0.85). The analysis of other APs in other traces revealed similar results, indicating that channels responsible for the after-hyperpolarization of APs were not altered. Thus, these data indicate that several fundamental parameters of APs are altered in EE mice compared to control mice.

### Enriched Environment Tunes Neurons for Firing at Higher Frequencies

**FIGURE 4:**
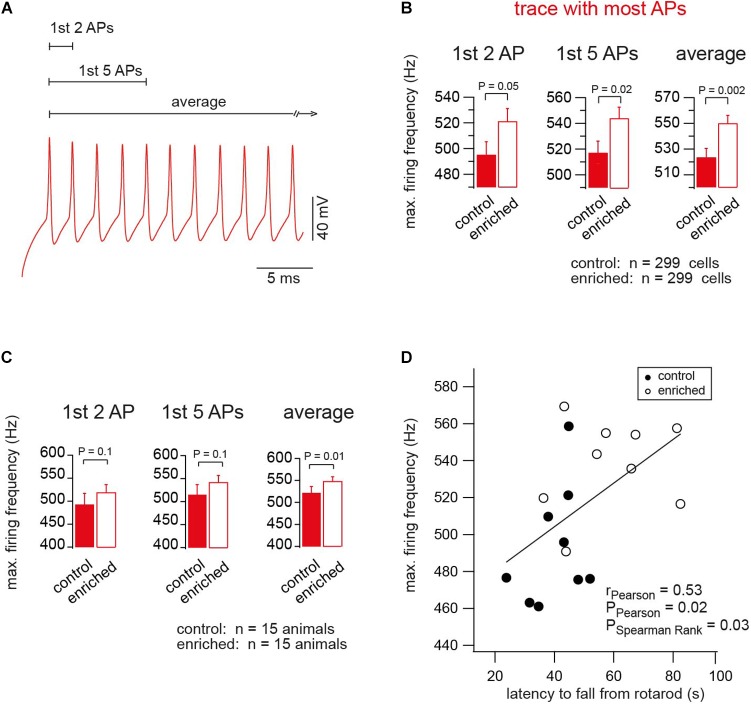
Enriched environment tunes neurons for firing at higher frequencies. **(A)** An example of the APs of the trace of maximum firing frequency (color-code as in Figure 2). **(B)** Average maximum firing frequency of neurons of EE and control mice, *n* = 299, 299 cells, based on the first two, first five APs, and average of all APs in the trace, in which most APs were fired, *P_t_*_-test_ = 0.05, 0.02, 0.002, respectively. **(C)** Average maximum firing frequency of neurons of EE and the corresponding control mice, *n* = 15, 15 mice. For the first two, first five APs and average of all APs in the trace, in which most APs were fired, *P_t_*_-test_ = 0.1, 0.1 and 0.01, respectively. **(D)** Correlation between the maximum firing frequency and the latency of the mouse to fall from rotarod, for control and EE mice, *n* = 9 and 9 mice, *r*_Pearson_ = 0.53, *P*_Pearson_ = 0.02, and *P*_SpearmanRank_ = 0.03.

The shortening of the AP half duration suggests that neurons are able to fire at higher frequencies after EE. We analyzed the maximum firing frequency of the first two APs (instantaneous frequency), the average of the first five APs, and average of all APs of the trace where most APs occurred (Figure 4A), and found that neurons of EE mice were able to reach higher firing frequencies than neurons of control mice (e.g., instantaneous frequency of 495.5 ± 9.8 and 521.6 ± 9.4 Hz, for control and EE mice, *n* = 299 and 299, respectively; *P_t_*_-test_ = 0.05; Figure 4B). We also analyzed the maximum firing frequency on a mouse-to-mouse basis corresponding to the analysis of the AP half duration in Supplementary Figure S1. Again, the maximum firing frequency showed a tendency to be larger in EE compared to control mice (*P_t_*_-test_ ≈ 0.1; Figure 4C). Finally, we observed a correlation between the maximum firing frequency per animal (i.e., the average from ∼20 granule cells) and the motor performance of the same animal measured on the same day (i.e., average latency of ∼10 trials on the rotarod; Figure 4D). In addition, there was an inverse correlation between maximum firing frequency and AP half duration (Supplementary Figure S3A). As a result, there was also a tendency of an inverse correlation between the latency on the rotarod and the AP half duration (Supplementary Figure S3B; see last paragraph of the discussion for a cautious interpretation of the correlations described here). These data indicate that the maximal firing frequency of granule cells is higher in EE compared to control mice.

## Discussion

Our data show that, upon EE, cerebellar granule cells of lobule IX of the cerebellum have altered AP parameters and can fire APs at higher frequencies. Thus, fundamental biophysical parameters of the neurons are influenced by the environment. Furthermore, the maximal firing frequency of granule cells correlated with the motor performance of the mice. This correlation does not provide a causal relationship between firing rate and behavior, but it is tempting to speculate that higher firing frequencies of cerebellar granule cells are beneficial for rapid sensory-motor integration.

### EE-Induced Changes in Action Potentials

We found that EE shortened the half-duration, increased the overshoot, and increased the threshold of APs. The observed effect is likely due to an alteration induced by EE on ion channels, so that voltage-gated potassium channels (K_v_) and sodium channels (Na_v_) have different densities and/or different properties (Keyvani et al., 2004). The observed shortening in the AP half duration could be explained by faster activating K_v_ channels. In fact, a regulatory effect of EE on potassium channels and particularly the regulatory subunit K_v_β1 has been reported (Need et al., 2003). The observed increase in the amplitude and the threshold of APs could be linked to a change in the density or properties of Na_v_ channels. Interestingly, an effect of EE on sodium channel Na_v_1.6 has been reported, in which EE decreased the amplitude of the ramp-induced persistent sodium current of the medium spiny neurons in the nucleus accumbens (Scala et al., 2018). Our data showing that the input resistance and the resting membrane potential were not altered upon EE is consistent with other work showing constant passive cell parameters during intrinsic homeostatic plasticity of cortical pyramidal neurons (Desai et al., 1999). Thus, the changes in ion channel properties and/or density upon EE are specific to channels shaping the AP and do not extend to channels setting the passive neuronal properties.

Our finding that EE alters AP properties adds to the emerging idea that AP properties are dynamically regulated. For example, it was recently shown that a direct modulation of presynaptic K_v_ channels in hippocampal mossy fiber boutons mediates a form of synaptic plasticity by activity-dependent release of arachidonic acid from the postsynaptic CA3 neurons (Carta et al., 2014). In addition, prominent alterations of AP half duration were observed during homeostatic plasticity induced in fast spiking interneurons (Miller et al., 2011). Furthermore, the AP half-duration of specific neurons in the amygdala and the cochlear nucleus was changed by fear extinction and noise exposure, respectively (Senn et al., 2014; Ngodup et al., 2015). Finally, altered neuronal activity in avian brainstem auditory neurons (Kuba et al., 2010) or hippocampal neurons (Grubb and Burrone, 2010) causes a rearrangement of the Na_v_ channels in the axon initial segment, contributing to the excitability and firing patterns of neurons (Evans et al., 2015; Kole and Brette, 2018). Our results are thus consistent with a scenario, in which EE alters neuronal activity, which in turn induces plastic alterations in ion channels responsible for shaping the AP waveform.

### Relation of Motor Performance and Firing Rate

Information can be coded as the average firing rate or as the temporal correlation of the exact time of the APs (Rieke et al., 1997). For example, vestibular, proprioceptive, and somatosensory information (Van Kan et al., 1993; Jörntell and Ekerot, 2006; Arenz et al., 2008) as well as the control of muscles (Adrian and Bronk, 1929) relies on rate coding. Our finding that improved motor performance correlates with AP frequency is therefore consistent with the idea that an increased bandwidth of firing accelerates information processing (reviewed in Delvendahl and Hallermann, 2016). Particularly, the increase in firing frequency of granule cells guarantees the precision of information transfer from the granule cell level to the Purkinje cell level, assuring a more precise pace-making role for Purkinje cells, which is critical in motor coordination (Walter et al., 2006).

We observed both a shortening of the AP half duration and an increase in the maximal firing frequency upon EE. There was an inverse correlation between maximum firing frequency and AP half duration (Supplementary Figure S3A). Such inverse correlation has also been observed within vestibular nucleus neurons (Gittis et al., 2010), across cell types (Carter and Bean, 2009), and across different species (Wang et al., 2016). As expected from the correlation between the latency on the rotarod and the maximal firing frequency (Figure 4D), we therefore also observed a tendency for an inverse correlation between the latency on the rotarod and the AP half duration (Supplementary Figure S3B).

Thus, our data provide support to the idea that the duration of the AP and the maximum frequency of firing are related to the speed of sensory-motor information processing. Interestingly, action potential kinetics were recently shown to correlate with another behavioral parameter (the intelligence quotient; IQ) in humans (Goriounova et al., 2018). However, several caveats should be considered regarding the relation between behavior and biophysical properties of neurons. (1) The correlations, which we observed here between performance on the rotarod and AP firing in granule cells, is statistically significant but not very strong. (2) The correlations do not imply causal relationships. (3) Our results were obtained in acute brain slices and future studies in freely behaving animals need to confirm the correlation between behavior and neuronal firing patterns *in vivo*. (4) Several other neuronal factors changing upon EE could contribute to the improved motor performance, such as myelination (Szeligo and Leblond, 1977; McKenzie et al., 2014), neuronal density (Kempermann et al., 1997), and dendritic and spine morphology (Volkmar and Greenough, 1972; Restivo et al., 2005). (5) Other parts of the nervous system (e.g., other types of neurons, other cerebellar lobules, the motor cortex, and the vestibular and proprioceptive systems) could change and cause the improved motor performance. (6) Factors independent of the nervous system (e.g., muscle strength and body weight) could underlie the improved rotarod performance. Yet, independent of the difficult question of the relation between behavior and neuronal biophysical properties, our data convincingly demonstrate that fundamental parameters such as AP duration and maximum firing frequency are influenced by the environment.

## Ethics Statement

All experiments were approved in advance by the Institutional Ethics Committees and animals were treated in accordance with the European (EU Directive 2010/63/EU, Annex IV for animal experiments), national, and Leipzig University guidelines.

## Author Contributions

AE and SH designed the study, analyzed the data, and wrote the manuscript. AE performed the experiments. All authors approved the final version of the manuscript.

## Conflict of Interest Statement

The authors declare that the research was conducted in the absence of any commercial or financial relationships that could be construed as a potential conflict of interest.

## Supplementary Material

The Supplementary Material for this article can be found online at: https://www.frontiersin.org/articles/10.3389/fncel.2019.00289/full#supplementary-material

FIGURE S1 Statistical analysis of AP half duration considering the average value for each mouse instead of the average value for each cell. The average AP half duration of 15 EE and 15 control mice is shown; (orange: trace at current threshold; cyan: trace with 60-pA-current injection; red: trace with maximum APs fired). From left to right, analysis of first AP, first five APs, and average of all APs, respectively. All the *P*-values shown are from Student’s *t*-test.

FIGURE S2 Constancy of action potential half duration between different age groups. **(A)** Average AP half duration of control mice of different age groups (*n* refers to the number of cells). **(B)** Histogram showing the distribution of the half duration of all APs recorded.

FIGURE S3 Correlation between behavior and electrophysiology. **(A)** Correlation between maximum firing frequency and AP half duration, for control and EE mice, *n* = 9, 9 mice, *r*_Pearson_ = 0.70, *P*_Pearson_ = 0.0007, *P*_SpearmanRank_ = 0.0002. **(B)** Correlation between AP half duration and latency to fall from rotarod, for control and EE mice, *n* = 9, 9 mice, *r*_Pearson_ = 0.43, *P*_Pearson_ = 0.07, *P*_SpearmanRank_ = 0.04.
