# Supplementary figures and images for: Enriched Environment Shortens the Duration of Action Potentials in Cerebellar Granule Cells
Source: Front Cell Neurosci. 2019 Jul 16;13:289. doi: 10.3389/fncel.2019.00289 (PMC6646744; doi:10.3389/fncel.2019.00289)

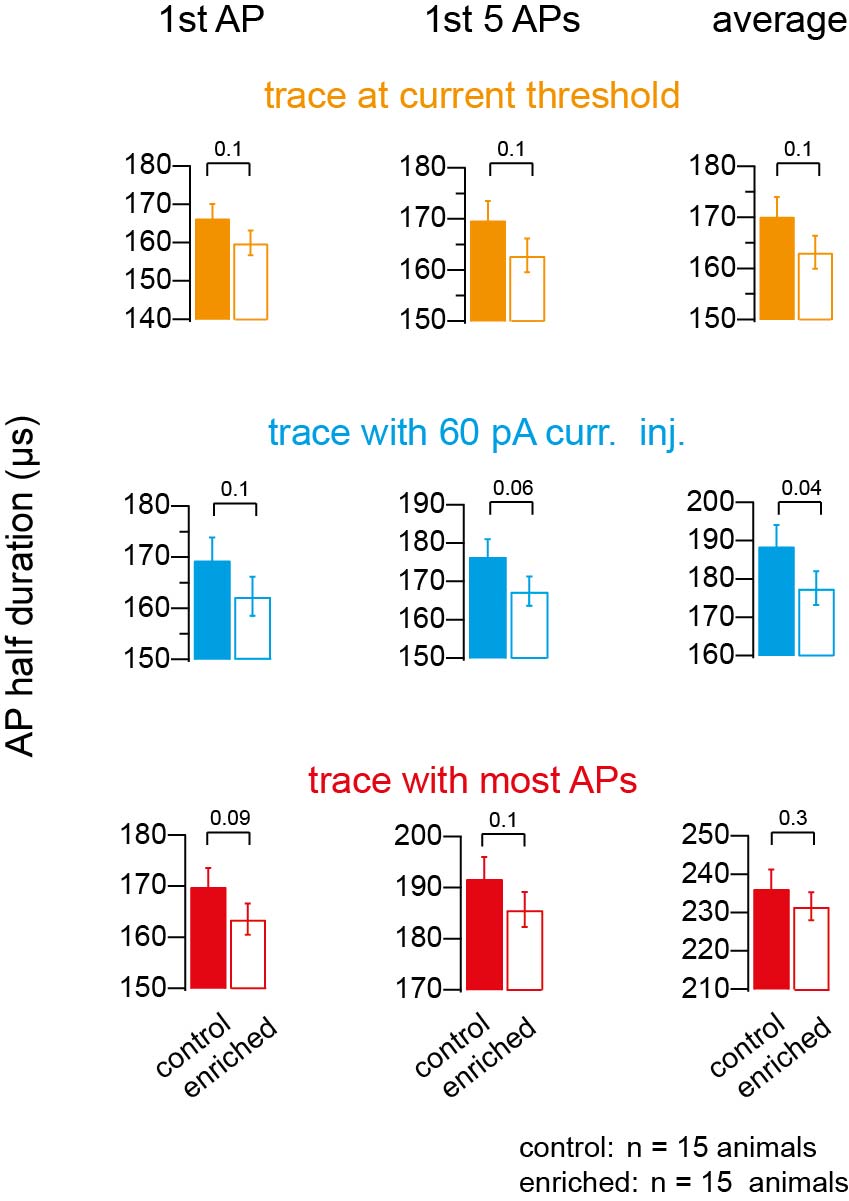

Supplement: FIGURE S1 — Statistical analysis of AP half duration considering the average value for each mouse instead of the average value for each cell. The average AP half duration of 15 EE and 15 control mice is shown; (orange: trace at current threshold; cyan: trace with 60-pA-current injection; red: trace with maximum APs fired). From left to right, analysis of first AP, first five APs, and average of all APs, respectively. All the P-values shown are from Student’s t-test. [file Image_1.jpg]

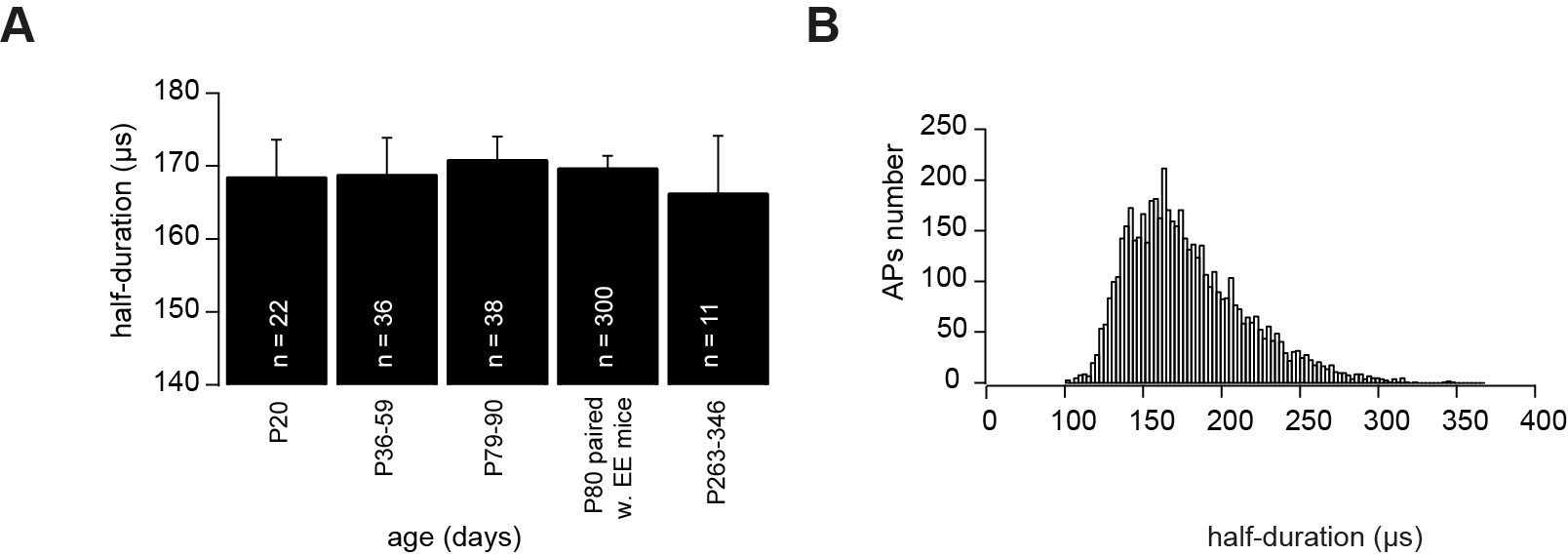

Supplement: FIGURE S2 — Constancy of action potential half duration between different age groups. (A) Average AP half duration of control mice of different age groups (n refers to the number of cells). (B) Histogram showing the distribution of the half duration of all APs recorded. [file Image_2.jpg]

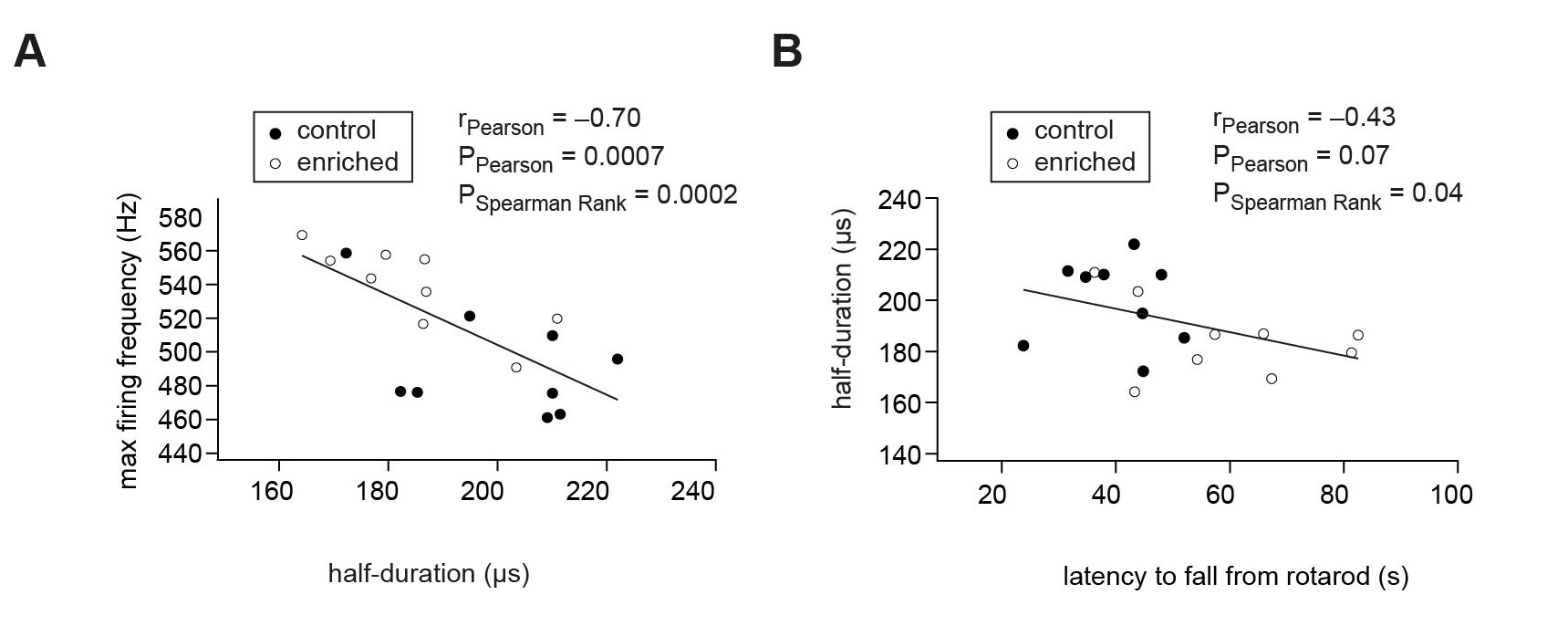

Supplement: FIGURE S3 — Correlation between behavior and electrophysiology. (A) Correlation between maximum firing frequency and AP half duration, for control and EE mice, n = 9, 9 mice, rPearson = 0.70, PPearson = 0.0007, PSpearmanRank = 0.0002. (B) Correlation between AP half duration and latency to fall from rotarod, for control and EE mice, n = 9, 9 mice, rPearson = 0.43, PPearson = 0.07, PSpearmanRank = 0.04. [file Image_3.jpeg]
